# Supplementary material for: Patterns of Intron Gain and Loss in Fungi
Source: PLoS Biol. 2004 Nov 30;2(12):e422. doi: 10.1371/journal.pbio.0020422 (PMC532390; doi:10.1371/journal.pbio.0020422)
Supplement: Table S1 — Also available at http://genes.mit.edu/NielsenEtAl/. (4.3 MB ZIP). [file pbio.0020422.st001.zip › NielsenEtAl/html/1193.html]

AN3199.1.NCU00809.1.MG05889.1.FG03272.1


```
 CLUSTAL W (1.82) Multiple Sequence Alignments - Introns Inserted


Sequence 1: AN3199.1	576 aa
Sequence 2: FG03272.1	535 aa
Sequence 3: NCU00809.1	547 aa
Sequence 4: MG05889.1	546 aa
Alignment Length: 607 aa
Number Identitical Residues: 268 aa
Alignment Score (without introns) 13913


MG05889.1 	MASSGDDKMATPLGDEKQKQVQADSMPENHNDAGQATALAEDVAAAQYSPWTASMWRLYA
NCU00809.1	------------MAHSINEKEAMGAHDDAFPESDITVQLAHDVDNTKYSPWSKSMFRLYL
FG03272.1 	------------MDSKADEKAGF-EHGAEYQDSHVAAQVAHDVQNQNLSPWTPSMFRLYL
AN3199.1  	------------MADEKTVQPSA-EQEAVGEAEEVR--VAHDALNAKYSPLTWSMFRLYL
          	            :  .   :                  :*.*.   : ** : **:*** 

MG05889.1 	VLACAYLCGCL0NGYDGSLMGGLNGMDSYRDYFGT~QVDGSSTGLTFAMYNIGGIAATAF
NCU00809.1	VLACAYLCGCL~NGYDGSLMGGLNGMKAYQNYFHM2STAGSGTGLVFAMYNIGSVAAVFF
FG03272.1 	VLACAYLCGCL0NGYDGSVMGGINGMDAYQHYFNM2SSEGSSTGIVFAMYNIGSIAAVFF
AN3199.1  	CLIIPYLCGTL~NGYDGSLMGGLNAMETYLDFFNM2ETSGSSTGIVFALYNIGSIPAVFF
          	 *  .**** * ******:***:*.*.:* .:*   .  **.**:.**:****.:.*. *

MG05889.1 	TGPVNDWFGRRWGMWIGAAI0IIIGTGVQAPAETGQRFLAGRFVLGFGVSFCCVSAPCYV
NCU00809.1	TAPVNDWFGRRWGMFTGALV~IIIGTCVQATTHTKSQFLGGRFVLGFGVSFCCVSAPCYV
FG03272.1 	TAPVNDWFGRRWGMFTGAAI~VIIGTCVQATSTNRGHFLGGRFILGFGVSFCCVSAPCYV
AN3199.1  	TGPVNDYWGRRCGMFVGALI~IVIGTCIQSPSVNRGMFLAGRFILGFGVSFCCVSAPCYV
          	*.****::*** **: ** : ::*** :*:.: .   **.***:****************

MG05889.1 	SEIAHPVWR-------------------GTLTGLYNCTW~YIGAILASWVTYAVAETMKG
NCU00809.1	SEMAHPKWR-------------------GTLTGLYNTTW~YIGSIIASWVVYGCSYIENN
FG03272.1 	SEMAHPTWR-------------------GTITGLYNCTW~YIGSIIAGWVVFGCSYLGKE
AN3199.1  	SEMAHPAWRYAPIFEGLSTKEILTVVSRGTITGLYNCTW2YIGSILASWVVYGCSQLDNA
          	**:*** ** :.  .. ::..  :  : **:***** ** ***:*:*.**.:. :   : 

MG05889.1 	SLDAWRIPVWCQLVTSGFVLIFALFLPESPRWLMANDRYEEAQRVLAKYHGEGNPEHPLV
NCU00809.1	KD-AWRIPIWCQMITSGIVCLGVFWLPESPRWLVAQDRYEEAAKILATYHGEGRLDHPLV
FG03272.1 	NDIAWRVPIWCQMVTSGFVVIFVLFLPESPRWLIAQDRVEDAVKVLAKYHGEGDPNHPMV
AN3199.1  	N--SFRIPIWCQLISSALVVLGVWFIPESPRWLMAQDRAEDAAKILTRYHGENDPDHPLV
          	.  ::*:*:***:::*.:* : . ::*******:*:** *:* ::*: ****.  :**:*

MG05889.1 	LLQLKEMTMQITQDGSDKRWWDYSALFNTRAHRRRMICVLGMGVFGQASGNSLS~SYYMA
NCU00809.1	QLQLKEMANQISSEASDKKWYDYHELWNTHSARRRLICVLGMACFGQVSGNSLS~SYYMV
FG03272.1 	IMQIKEMTHQIASDATDKSWWDYRGLWNSHSARRRLIGVLGMAVFGQVSGNSLS2SYYLP
AN3199.1  	HLQLKEMQQSIATDASDKKWWDYRELYTGHSARRRLICVLGMACFGQISGNSVT~SYYLP
          	 :*:***  .*: :.:** *:**  *:. :: ***:* ****. *** ****:: ***: 

MG05889.1 	AMLKTAGITEQGRVLAFNATNPMVSFIGAVLGARMTDVVGRRPLLLSTIVFASVCFAIIT
NCU00809.1	NMLKSAGITDEHKVLALNGINPALSLIGAVMGARMTDVVGRRPLLLYTIVFSSTCFAIMT
FG03272.1 	VMMKYAGIVEEKKVLALNGVNPVLCFFGAILGARMTDVIGRRPLLLYSIIFCSCCFAIIT
AN3199.1  	VMLENAGIVSESRKLLFNGIYPPLSLIGAVVGARMTDTIGRRPLLIYSLLFCSVAFAIIT
          	 *:: ***..: : * :*.  * :.::**::******.:******: :::*.* .***:*

MG05889.1 	GTTKLATE--------------DPSNTAAGNATVAFIFIFGVVFSFGWTPLQSMYIAETL
NCU00809.1	GTSKLAMPGHVGNLDDADTQALTPSQRAAANTTIAFIFIFGIVFSFGWTALQSMYIAETL
FG03272.1 	GTSKLSLD--------------DRDNSSAANATVAMIFIFGIVFSFGWTPLQSAYIAECL
AN3199.1  	GTSKLATD--------------DPTNTAAANTTIAFIYLFGIVFSFGWTPLQSMYIAETL
          	**:**:                   : :*.*:*:*:*::**:*******.*** **** *

MG05889.1 	PTETRAKGTAIGSLASACASTVLQYSAGPAFESISYYYYIVFVFWDLFEGVIIYF2FWPE
NCU00809.1	PTATRAKGTAVGNFVSAASSVVLQYSSGPAFEKIGYYFYLVFVFWDLIEGVLIFF~YFPE
FG03272.1 	STDTRAKGTAVGNLASSIASTIIQYSSGPAFQKIGYHFYLVFVFWDLFEAVIIWF~FFPE
AN3199.1  	TTTTRAKGTAVGNLASSIASTIIQYSSGPAFKDIQYYFYLVFVFWDLIEIVIMYF~YFPE
          	.* *******:*.:.*: :*.::***:****:.* *::*:*******:* *:::* ::**

MG05889.1 	TKDRTLEELEEVFQAPNPVKKSLEKRDTHTVLNTLNANNDKM~LSV--------------
NCU00809.1	TKDRTLEELEEVFSAPNPVKKSLEKRSAQTVLNTMGAPDDEK~VTI--------------
FG03272.1 	TKDRTLEELSEVFEAPNPVKKSLQKRDANTVMNTMNVAGDEK~LTGDV------------
AN3199.1  	TKDRTLEELEEVFSAPNPVKRSLVKRDAATVLNTMQVEQREL0VYIPATMGTTYSRRSEF
          	*********.***.******:** **.: **:**: .   :  :    : .:: :  :. 

MG05889.1 	--------------
NCU00809.1	--------------
FG03272.1 	--------------
AN3199.1  	LWQSLSSANDLRPA
          	  .: :::..  .:
```
